# Quercetin and Silybin Decrease Intracellular Replication of *Piscirickettsia salmonis* in SHK-1 Cell

**DOI:** 10.3390/ijms26031184

**Published:** 2025-01-29

**Authors:** Mick Parra, Katherin Izquierdo, Meraiot Rubio, Antonia de la Fuente, Mario Tello, Brenda Modak

**Affiliations:** 1 Laboratory of Natural Products Chemistry and Their Applications, Centre of Aquatic Biotechnology, Faculty of Chemistry and Biology, University of Santiago of Chile, Santiago 9170022, Chile; mick.parra@usach.cl (M.P.); meraiot.rubio@usach.cl (M.R.); 2 Laboratory of Basic and Applied Microbiology, Faculty of Chemistry and Biology, University of Santiago of Chile, Santiago 9170022, Chile; katherin.izquierdo@usach.cl; 3 Laboratory of Bacterial Metagenomic, Centre of Aquatic Biotechnology, Faculty of Chemistry and Biology, University of Santiago of Chile, Santiago 9170022, Chile; antonia.delafuente@usach.cl (A.d.l.F.); mario.tello@usach.cl (M.T.)

**Keywords:** *Piscirickettsia salmonis*, SHK-1, silybin, quercetin, flavonids, intracellular replication

## Abstract

*Piscirickettsia salmonis* is the pathogen that has most affected the Chilean salmon industry for over 30 years. Considering the problems of excessive use of antibiotics, it is necessary to find new strategies to control this pathogen. Antivirulence therapy is an alternative to reduce the virulence of pathogens without affecting their growth. Polyphenolic compounds have been studied for their antiviral capacity. In this study, the capacity of quercetin and silybin to reduce the intracellular replication of *P. salmonis* in SHK-1 cells was evaluated. For this, three different infection protocols in Salmon Head Kidney-1(SHK-1) cells were used: co-incubation for 24 h, pre-incubation for 24 h prior to infection, and post-incubation for 24 h after infection. In addition, the effect of co-incubation in rainbow trout intestinal epithelial cells (RTgutGC) and the effect on the phagocytic capacity of SHK-1 cells were evaluated. The results obtained showed that quercetin and silybin decreased the intracellular replication of *P. salmonis* in SHK-1 cells when they were co-incubated for 24 h; however, they did not have the same effect in RTgutGC cells. On the other hand, both compounds decreased the phagocytosis of SHK-1 cells during co-incubation. These results are promising for the study of new treatments against *P. salmonis*.

## 1. Introduction

The salmon industry occupies an important place in the Chilean economy, positioning the country as the second largest producer of salmonids worldwide, after Norway [1]. The three species with the highest production, Atlantic salmon, coho salmon, and rainbow trout, are affected by *Piscirickettsia salmonis*. This bacterium was responsible for 50% of pathogen-associated mortalities in Atlantic salmon and coho salmon and 7% of mortalities in rainbow trout in 2022 [2]. This bacterium has affected the national salmon industry for more than 30 years, generating annual losses of over USD 700 million [3]. In Chile, the most commonly used treatments for the control of *P. salmonis* are antibiotics, mainly florfenicol. In 2022, 341.5 tons of antibiotics were utilized, 97.7% of which were used in the seawater phase of salmonids, 91.3% of which were used to treat piscirickettsiosis [4]. The massive use of antibiotics has generated an increase in environmental pollution as a result of the incomplete metabolization of antibiotics by fish, which causes the accumulation of traces of antibiotics or their sub-products in the urine and feces of salmon in the marine sediment [5,6]. Another negative impact of the generalized use of antibiotics in aquaculture is related to the international market, since Chilean products have been negatively evaluated due to the traces of antibiotics present in the muscle, making them a less attractive product for marketing. Globally, the increase in the antibiotic resistance of pathogenic bacteria affecting humans and animals due to the intensive and uncontrolled use of antibiotics in livestock industry, aquaculture, human health treatments, and other areas has led the FAO (Food and Agriculture Organization of the United Nations) and WHO (World Health Organization) to promote reductions in administration in both human and animal industries. In recent decades, due to the increase in antibacterial resistance, the effect of natural products as an alternative to the use of antibiotics has been studied, especially against multi-resistant Gram-positive and Gram-negative bacteria [7]. Some of the most studied natural compounds are flavonoids. Several studies have shown the antibacterial capacity of various flavonoids. For example, compounds such as naringenin and pinocembrin have been widely studied for their antibacterial activity [8,9,10] in bacteria such as *Enterobacter cloacae*, *Escherichia coli*, and *Klebsiella pneumoniae* [11]. The effects of other compounds, such as 3′-O-methyldiplacol, against *Bacillus cereus*, *Bacillus subtilus*, *Enterococcus faecalis*, *Listeria monocytogenes*, and *Staphylococcus aereus* have been reported [12]. Additionally, the effects of quercetin-3-O-α-L-rhamnoside against *Salmonella typhimurium* [13], (-)—epicatechins against *Helicobacter pylori* isolates NCTC 11637 and 26695 [14], and semilycoisoflavone B against vancomycin-resistant *Enterococcus* sp. [15], among many others, have also been reported. The antibacterial effect of natural compounds is not the only capacity reported in these molecules. Natural compounds can also act by inhibiting or blocking the expression of virulence factors present in pathogenic bacteria without interfering with processes essential for the viability of the bacteria. The use of natural compounds in doses where this activity is present is called antivirulence therapy and constitutes an alternative to conventional antimicrobial-based therapy [16]. Among the factors and processes that can be inhibited through antivirulence therapy are biofilm formation [17,18], the expression of molecules involved in quorum sensing [19], secretion systems [19], motility [20,21], and bacterial adhesion [22,23]. *P. salmonis* is able to survive and replicate within replicative vacuoles in phagocytic cells such as macrophages and monocyte-type cells [24] and persists inside phagocytic cells by evading phagosome–lysosome fusion [25] through the activation of the type IV secretion system. Furthermore, this bacterium has several virulence factors which favor its pathogenicity, such as biofilm formation [26,27,28], the production of proteolytic enzymes [29], adherence to the cell surface through expression of type IV pili (T4P) [30], and the expression of flagellin [31,32], among others. Previous studies carried out in our laboratory with a group of polyphenols with known antibacterial activity evaluated their antibacterial capacity on *P.salmonis*, where quercetin and silybin showed the best results. Based on that, in this study, the ability of the polyphenolic compounds quercetin and silybin to decrease the intracellular replication of *P. salmonis* in the SHK-1 cell line at sub-inhibitory concentrations was evaluated as an alternative to antibacterial treatments against *P. salmonis*. The results obtained showed that both compounds have the ability to decrease the intracellular replication of *P. salmonis* in SHK-1 cells, but only when they are co-incubated with the bacteria, suggesting that the interaction between the compound, the bacteria, and the cell is necessary for the antivirulence effect.

## 2. Results

### 2.1. Antibacterial Effect of Silybin and Quercetin

The analysis of the antibacterial capacity of quercetin and silybin on two *P. salmonis* isolates in cell-free medium was performed by calculating the minimum inhibitory concentration (MIC) and the concentration that inhibits 50% of bacterial growth (IC_50_). The results obtained showed that quercetin has a greater antibacterial effect on *P. salmonis* than silybin. The MIC values determined for quercetin were 38.6 µg/mL for isolate CGR02 and 62.7 µg/mL for isolate 12201, while the IC_50_ values were 26.8 µg/mL and 30.2 µg/mL, respectively. On the other hand, the MIC values for silybin were 134.3 µg/mL for isolate CGR02 and 132.5 µg/mL for isolate 12201, while the IC_50_ values were 71.0 µg/mL and 74.0 µg/mL, respectively (Table 1).

### 2.2. Effect of Quercetin and Silybin on the Intracellular Replication of P. salmonis in SHK-1 Cells

In the search for treatments that affect the intracellular replication of *P. salmonis* in cell culture without an antibacterial effect, it was decided to use sub-inhibitory concentrations of quercetin and silybin, IC_50_ (Q in the case of quercetin and S in the case of silybin), ½ IC_50_ (Q/2 in the case of quercetin and S/2 in the case of silybin), and ¼ IC_50_ (Q/4 in the case of quercetin and S/4 in the case of silybin) (Table 2).

**Figure 1:**
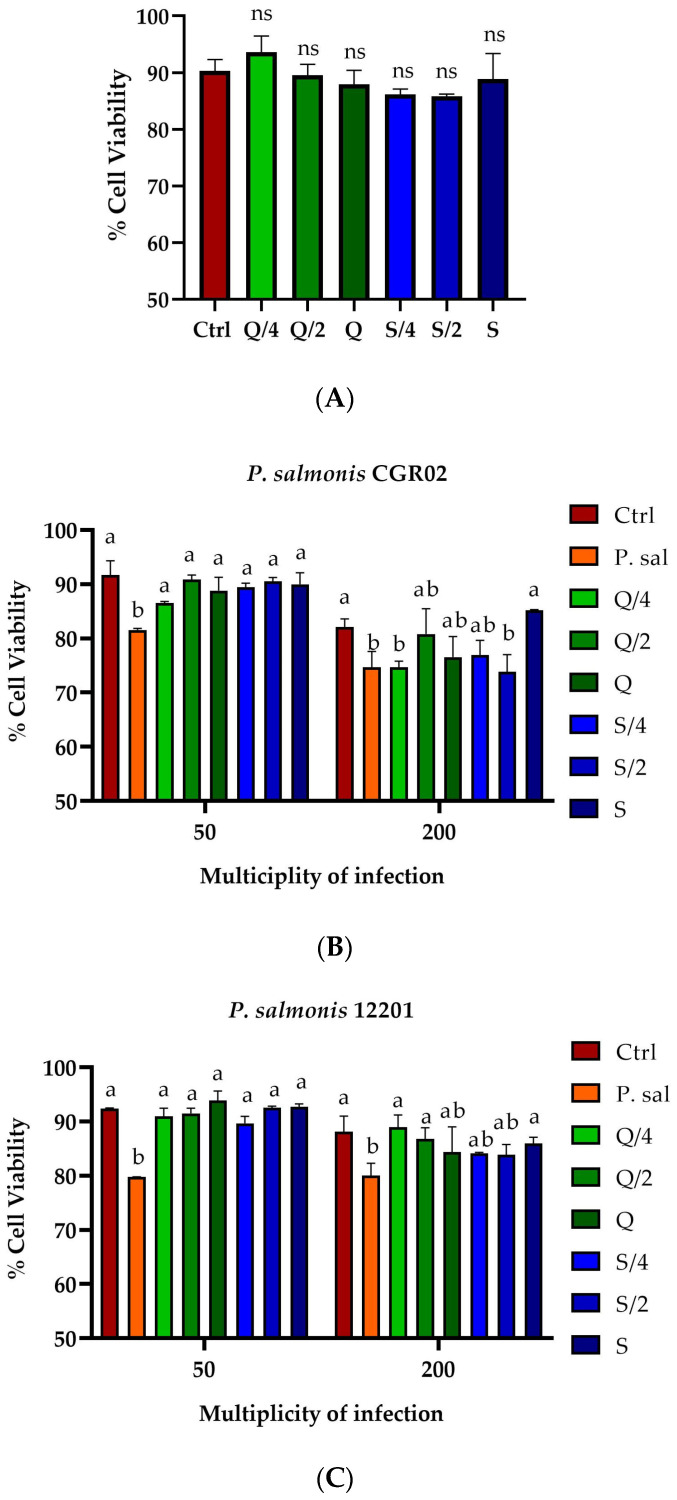
Evaluation of the cytotoxic effects of quercetin, silybin, and *P. salmonis* in SHK-1 cells. (**A**) Viability of SHK-1 cells after 24 h of incubation with quercetin and silybin. (**B**) Viability of SHK-1 cells after 14 days of infection with *P. salmonis* CGR02 and co-incubation with quercetin and silybin for 24 h. (**C**) Viability of SHK-1 cells after 7 days of infection with *P. salmonis* 12201 and co-incubation with quercetin and silybin for 24 h. Ctrl = control cell without infection. P. sal = control cells only infected with *P. salmonis.* Q, Q/2, and Q/4 correspond to the cells co-incubated with 27, 14, and 7 µg/mL of quercetin. S, S/2, and S/4 correspond to the cells co-incubated with 68, 34, and 17 µg/mL of silybin. ns = no statistically significant differences. Different letters mean statistically significant differences.

The effect of 24 h of incubation of the three concentrations of quercetin and silybin on the viability of SHK-1 cells was evaluated. The results showed that none of the three concentrations of quercetin and silybin had cytotoxic effects on SHK-1 cells after 24 h of incubation (Figure 1A). Subsequently, the co-incubation of these two compounds using sub-inhibitory concentrations to determine the effect on the intracellular replication of two *P. salmonis* isolates in SHK-1 cells was evaluated. The results showed that co-incubation of the three concentrations of quercetin decreased the cytotoxic effect of *P. salmonis* CGR02 when it was infected with an multiplicity of infection (MOI) of 50, increasing cell viability from 81% in cells infected only with *P. salmonis* CGR02 (P. sal) to approximately 86% with co-incubation of 7 μg/mL of quercetin (Q/4) and 90% with co-incubation of 14 and 27 μg/mL of quercetin (Q/2 and Q), statistically similar to what was obtained for the control group (Ctrl). In the co-incubation with silybin, the three concentrations used, 17 μg/mL (S/4), 34 μg/mL (S/2), and 68 μg/mL (S), also decreased the cytotoxic effect of *P. salmonis* CGR02, increasing cell viability nearly 90%, similar to what was observed for the control group (Ctrl) (Figure 1B). When the amount of bacteria was increased to an MOI of 200, the three concentrations of quercetin did not show a reduction in the cytotoxicity of *P. salmonis* in SHK-1 cells, obtaining a viability percentage between 75 and 80%, while the control cells infected only with *P. salmonis* CGR02 (P. sal) presented a viability of 75% (Figure 1B). On the other hand, only incubation with the highest concentration of silybin, 68 μg/mL (S), increased cell viability to 85% compared to the 75% viability of cells infected with *P. salmonis* CGR02 (Figure 1B). The co-incubation experiments were repeated, but the cells were infected with *P. salmonis* 12201 using an MOI of 50. The results obtained with the three concentrations of quercetin, 7, 14, and 27 μg/mL (Q/4, Q/2 and Q), showed that the cytotoxicity of *P. salmonis* 12201 in the cells decreased, increasing the viability of the cells infected with *P. salmonis* 12201 from 80% in the case of control cells infected only with *P. salmonis* (P. sal) to cell viability values greater than 90%, without statistical difference from the uninfected control cells (Ctrl) (Figure 1C). The co-incubation with the three concentrations of silybin, 17 μg/mL (S/4), 34 μg/mL (S/2), and 68 μg/mL (S), also decreased the cytotoxic effect of *P. salmonis* 12201, increasing cell viability to nearly 90%, similar to the value obtained for the control group (Ctrl) (Figure 1C). When the amount of bacteria was increased to an MOI of 200, it was observed that only 7 and 14 μg/mL (Q/4 and Q/2) of the quercetin reduced the cytotoxicity of *P. salmonis* in SHK-1 cells, increasing cell viability to 90% and 85%, respectively, compared to the cells infected only with *P. salmonis* 12201 (80%). The co-incubation with silybin, again at a concentration of 68 μg/mL (S), decreased the cytotoxic effect of *P. salmonis*, increasing cell viability to nearly 90%, similar to what was the obtained in the uninfected control group (Ctrl) (Figure 1C).

**Figure 2:**
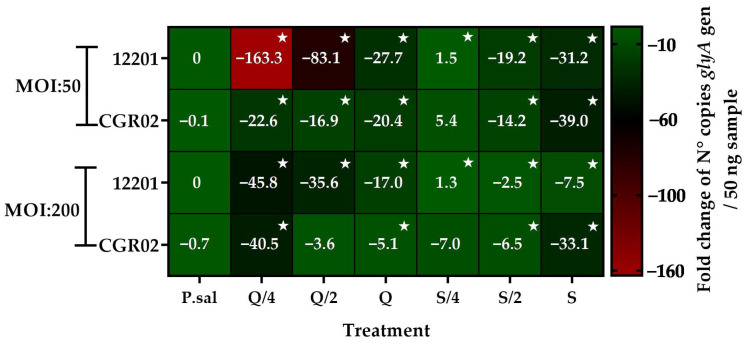
Intracellular replication of different *P. salmonis* isolates in SHK-1 cells assessed by detection of the *glyA* gene at 7 and 14 days post-infection. Cells were infected with *P. salmonis* CGR02 and *P. salmonis* 12201 at MOIs of 50 and of 200 and co-incubated with quercetin and silybin. C+ = Control cells infected with *P. salmonis*. Q/4 = 7 μg/mL. Q/2 = 14 μg/mL. Q = 27 μg/mL. S/4 = 17 μg/mL. S/2 = 34 μg/mL. S = 68 μg/mL. Stars mean statistically significant differences between treatments and a control *p* value < 0.05.

Considering the results on the viability of SHK-1 cells, the intracellular replication of *P. salmonis* 12201 and *P. salmonis* CGR02 was evaluated by quantitative Polymerase Chain Reaction (qPCR). The results showed that the co-incubation with the three different concentrations of quercetin decreased the intracellular replication of *P. salmonis* 12201 (MOI of 50). The concentration of 7 μg/mL (Q/4) managed to decrease the bacterial load (evaluated by the number of copies of the *glyA* gene) by 160 times, while with 14 μg/mL (Q/2) and 27 μg/mL (Q) concentrations, the bacterial load was reduced by 80 and 27 times with respect to the control cells infected with *P. salmonis* (C+). In the case of co-incubation with silybin, only the two highest concentrations decreased the bacterial load by 20 and 30 times, respectively. When the infection was carried out using an MOI of 200, similar results were observed, but with a lower magnitude. Thus, it was possible to detect reductions in the bacterial load of 45, 36, and 17 times in the co-incubations with quercetin at 7, 14, and 27 μg/mL, respectively. Silybin also reduced the bacterial load of *P. salmonis*, but only by about 3 and 8 times when they were co-incubated at concentrations of 34 and 68 μg/mL, respectively (Figure 2).

In the infection performed with *P. salmonis* CGR02 at an MOI of 50, the co-incubation with the three concentrations of quercetin analyzed in this study decreased the bacterial load by approximately 20 times. In the case of silybin, similar results to those seen with *P. salmonis* 12201 were observed, detecting 15- and 40-fold reductions in the bacterial load with the co-incubations of 34 μg/mL (S/2) and 68 μg/mL (S), respectively. When the infection was carried with an MOI of 200, 40- and 5-fold reductions were detected in the co-incubations with 7 μg/mL (Q/4) and 27 μg/mL (Q) of quercetin, respectively. In the case of silybin, only the co-incubation with the two highest concentrations showed a decrease in the number of copies of the *glyA* gene, by about 7 and 33 times (Figure 2). 

**Figure 3:**
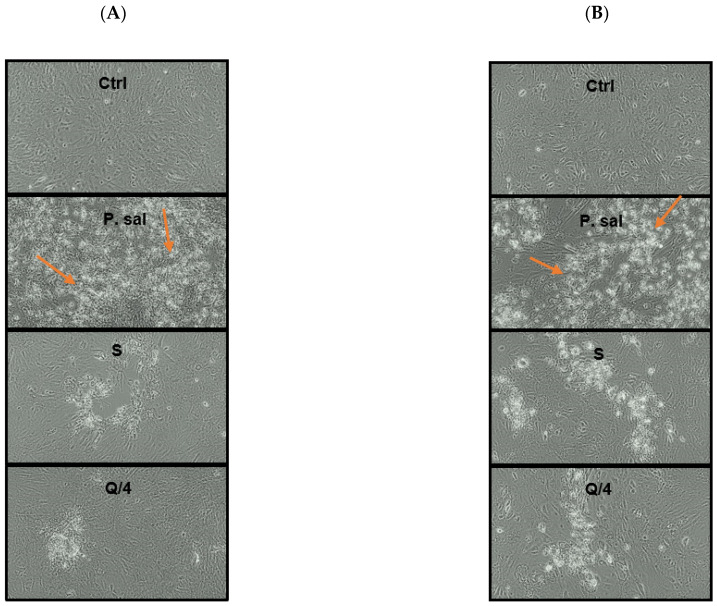
Visualization of the cytopathic effect of *P. salmonis* in SHK-1 cells co-incubated with silybin. Ctrl= control cells. P. sal = cells infected with *P. salmonis*. Q/4 = 7 μg/mL of quercetin. S = 68 μg/mL of silybin. (**A**) Infection performed with *P. salmonis* 12201. (**B**) Infection performed with *P. salmonis* CGR02. Arrows indicate cytopathic effect characteristic of *P. salmonis*.

The characteristic cytopathic effect of *P. salmonis* on SHK-1 cell infection was alsoaffected by quercetin and silybin treatments. Decreases in the cytopathic effects of *P. salmonis* 12201 (Figure 3A) and *P. salmonis* CGR02 (Figure 3B) were observed in co-incubations with the three concentrations of quercetin and silybin, which is related to the decrease in the cytotoxic effect of *P. salmonis* on SHK-1 cells and the decrease in bacterial replication detected by qPCR.

**Figure 4:**
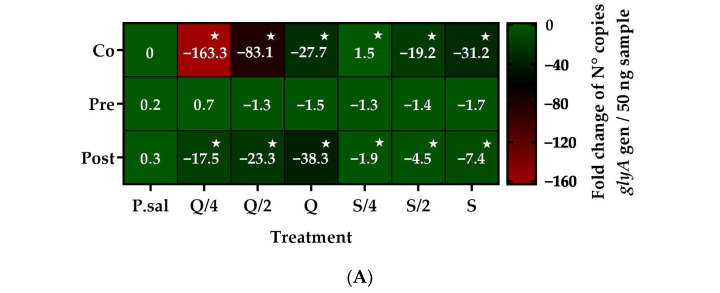

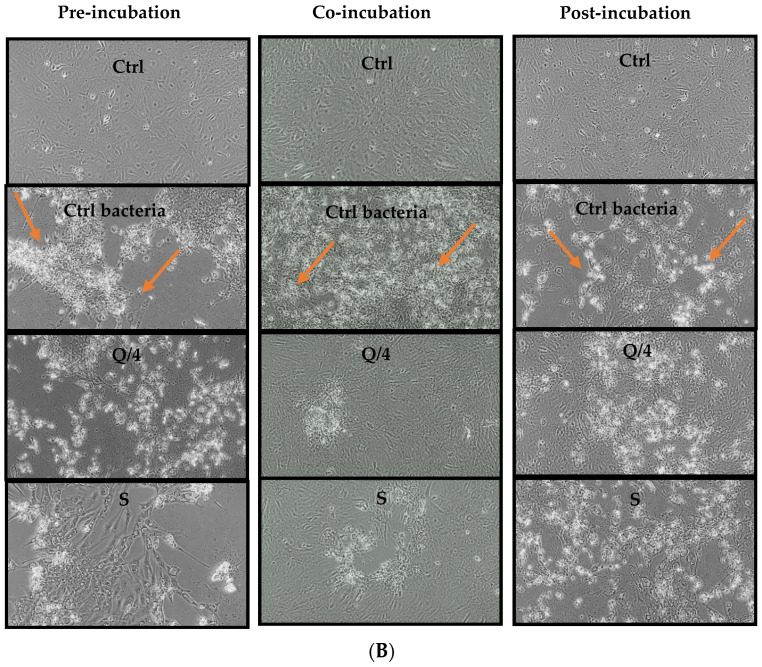
Intracellular replication of *P. salmonis* 12201 in -1 cells incubated with quercetin and silybin in different infection protocols. (**A**) Quantification of the bacterial load by detection of the *glyA* gene at 7 days post-infection. (**B**) Visualization of the cytopathic effect of *P. salmonis* 12201 in SHK-1 cells. Ctrl = Control cells without infection. P.sal = Control cells infected with *P. salmonis*. Q/4 = 7 μg/mL. Q/2 = 14 μg/mL. Q = 27 μg/mL. S/4 = 17 μg/mL. S/2 = 34 μg/mL. S = 68 μg/mL. The stars mean statistically significant differences between the treatments and a control *p* value < 0.05.

The results showed that the co-incubation with silybin and quercetin decreased the cytotoxic and cytopathic effects of *P. salmonis* on SHK-1 cells, as well as the intracellular replication of the bacteria. With these results, the ability to decrease the replication of *P. salmonis* was evaluated using pre- incubation and a post- incubation with *P. salmonis* 12201. The pre-incubation protocol with quercetin and silybin at their three concentrations using an MOI of 50 did not have a statistically significant effect on the bacterial load of *P. salmonis* (Figure 4A). On the other hand, the post-incubation of the compounds did have an effect in decreasing the bacterial load of *P. salmonis,* but to a lesser extent than co-incubation. Quercetin decreased the copy number of the *glyA* gene by approximately 18, 23, and 38 times at 7 μg/mL (Q/4), 14 μg/mL (Q/2), and 28 μg/mL (Q), respectively. Silybin decreased the copy number of the *glyA* gene by approximately 2, 5, and 7 times at 17 μg/mL (S/4), 34 μg/mL (S/2), and 68 μg/mL (S), respectively (Figure 4A). Similar results were obtained when representative images of the cytopathic effects of *P. salmonis* from the different incubation protocols were observed. For example, when *P. salmonis* was co-incubated with 7 μg/mL of quercetin (Q/4) and 68 μg/mL of silybin (S), it was possible to observe a considerable decrease in the cytopathic effect of *P. salmonis* on SHK-1 cells. However, the same concentrations of quercetin and silybin had a lower effect when they were post-incubated, while when they were pre-incubated they did not decrease the cytopathic effect of *P. salmonis* on SHK-1 cells (Figure 4B).

### 2.3. Effect on the Intracellular Replication of P. salmonis in RTgutGC Cells

**Figure 5:**
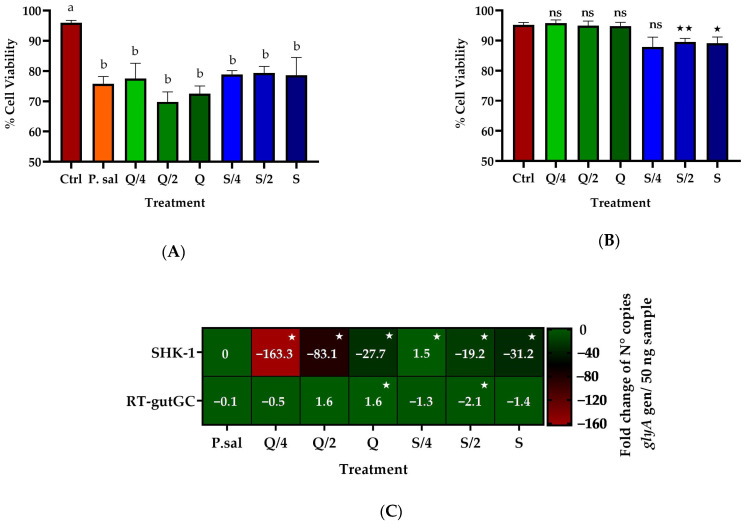

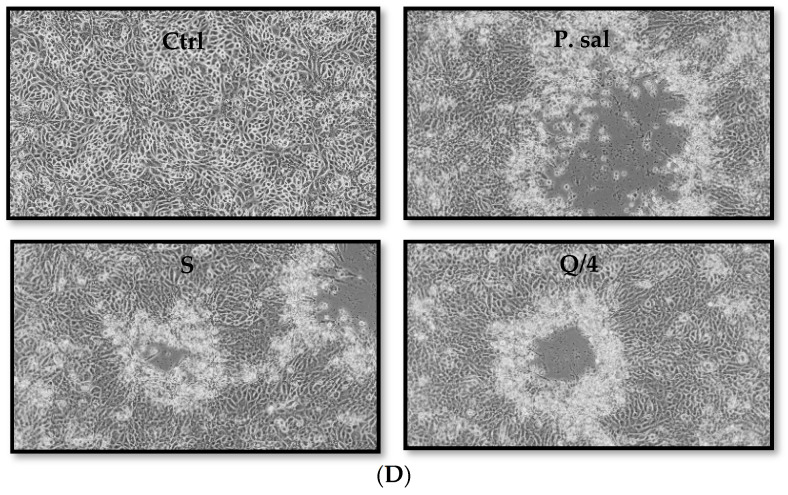
Intracellular replication of *P. salmonis* 12201 in RTgutGC cells incubated with quercetin and silybin. (**A**) Viability of RTgutGC cells after 24 h of incubation with quercetin and silybin. (**B**) Viability of RTgutGC cells after 14 days of infection with *P. salmonis* 12201 and co-incubation with quercetin and silybin for 24 h. (**C**) Intracellular replication of *P. salmonis* 12201 in RTgutGC cells assessed by detection of the *glyA* gene at 14 days post-infection. (**D**) Visualization of the cytopathic effect of *P. salmonis* 12201 in RTgutGC cells. Ctrl = control cells without infection. P. sal= control cells only infected with *P. salmonis.* Q, Q/2, and Q/4 correspond to the cells co-incubated with 27, 14, and 7 µg/mL of quercetin. S, S/2, and S/4 correspond to the cells co-incubated with 68, 34, and 17 µg/mL of silybin. Statistically significant differences were found with respect to the control. (* *p* value < 0.05, ** *p* value < 0.001, ns= not significant). Different letters mean statistically significant differences.

The ability of quercetin and silybin to decrease intracellular replication of *P. salmonis* was evaluated in a non-phagocytic cell line, for which the RTgutGC cell line was used. First, the cytotoxicity of incubation with quercetin and silybin after 24 h was evaluated. The results obtained showed that incubation with the three concentrations of quercetin (Q, Q/2, and Q/4) did not affect the viability of RTgutGC cells; however, a statistically significant decrease of approximately 5% in cell viability was obtained when they were incubated with the two highest concentrations of silybin (S and S/2) (Figure 5A). Subsequently, the effect of co-incubation of quercetin and silybin during an infection with *P. salmonis* 12201 at an MOI of 50 in RTgutGC cells was evaluated. The results showed that the cytotoxic effect that *P. salmonis* generates on the RTgutGC cell line was not diminished by any of the treatments. It was observed that the viability of the cells infected only with *P. salmonis* was 75%, similar to that observed in cells that were co-incubated with quercetin (Q, Q/2, and Q/4) and silybin (S, S/2, and S/4), while the viability of uninfected cells was approximately 95% (Figure 5B). On the other hand, when the bacterial load present in the different treatments was evaluated, none of the three concentrations of quercetin analyzed by co-incubation reduced the number of copies of the *glyA* gene, unlike what was observed in the SHK-1 cell line (Figure 5C). Similar results were observed with silybin, detecting only a slight decrease of about 2 times with the co-incubation of 34 μg/mL (S/2) (Figure 5C). Finally, the cytopathic effect of *P. salmonis* showed that there were no differences between the cells infected only with *P. salmonis* and the cells treated with the different concentrations of quercetin and silybin (Figure 5D).

### 2.4. Decreased Phagocytic Capacity of SHK-1 Cells

**Figure 6:**
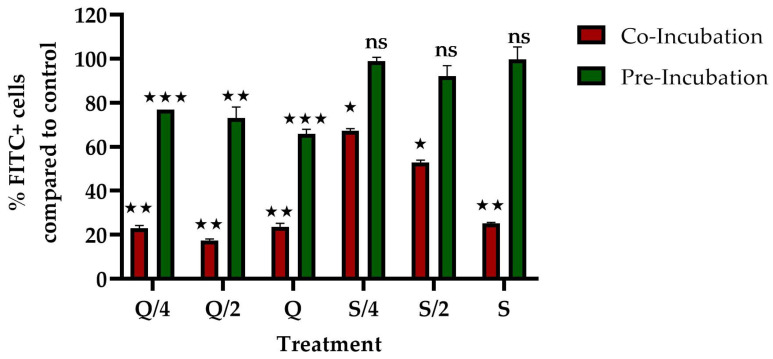
Phagocytosis assay using carboxylate-modified FluoSpheres in SHK-1 cells. Cells were pre-incubated and co-incubated with quercetin and silybin. Q/4 = 7 μg/mL. Q/2 = 14 μg/mL. Q = 27 μg/mL. S/4 = 17 μg/mL. S/2 = 34 μg/mL. S = 68 μg/mL. Stars mean statistically significant differences between treatments and the control. ns = no significant differences from control cells. * *p* value < 0.05, ** *p* value < 0.01, *** *p* value < 0.001.

Considering the results obtained for the co-incubation of quercetin and silybin with *P. salmonis* in the infection process on SHK-1 and RTgutGC cells, and the fact that these compounds were able to decrease the replication of *P. salmonis* only in phagocytic-type cells, the ability of quercetin and silybin to affect the phagocytosis process of SHK-1 cells was evaluated. The results showed that co-incubation of SHK-1 with the three concentrations of quercetin considerably decreased the phagocytic capacity of SHK-1 cells, detecting about 20% of Fluorescein isothiocyanate (FITC+) cells compared to control cells (Figure 6). The co-incubation with silybin presented a dose-dependent effect: with 17 μg/mL (S/4) of the compound, 65% of the cells were FITC+, while with 34 μg/mL (S/2), about 50% of the cells were FITC+. Finally, with 68 μg/mL (S), only about 25% of the cells were FITC+ (Figure 6). On the other hand, the effect of pre-incubation showed different results from those observed in the co-incubation experiments on the phagocytic capacity of SHK-1. Pre-incubation with quercetin had a dose-dependent effect on phagocytic capacity, but of a lower magnitude when compared to the effect of co-incubation. At 7 μg/mL (Q/4), about 77% of cells were FITC+ compared to control cells, while at 14 μg/mL (Q/2) about 70% were FITC+ cells and at 27 μg/mL (Q) about 65% of cells were FITC+. The pre-incubation with silybin had no effect on the phagocytic capacity of the cells, observing a percentage of FITC+ cells of 100% when compared to control cells (Figure 6).

## 3. Discussion

An alternative to the use of antibiotics or antimicrobials that inhibit the growth of bacterial pathogens is antivirulence therapy, which is based on inhibiting virulence factors of pathogenic bacteria, reducing their pathogenicity without affecting the growth of microorganisms [16]. Natural compounds such as flavonoids have been studied for their antivirulence capacity. It has been reported that flavonoid–protein interactions (enzymes, receptors, transporters, and transcription factors, among others) are phenomena that regulate the beneficial effects of these compounds on pathogenic bacteria [33]. In the case of *P. salmonis*, it has been reported that it possesses various virulence factors than can be affected using antivirulence therapy, such as the formation of biofilms, secretion of exotoxins, expression of proteins such as flagellin, and type IV pili (TP4), in addition to different adherence and invasion proteins [34], which are essential to the ability of *P. salmonis* to internalize itself in phagocytic cells and replicate inside them in replicative vacuoles [24]. For these reasons, in this study, the potential of quercetin and silybin to decrease the internalization and/or intracellular replication of *P. salmonis* at sub-inhibitory concentrations was evaluated. To achieve this objective, three different infection protocols were used: co-incubation, pre-incubation, and post-incubation.

The results obtained with the co-incubation protocol showed that quercetin and silybin decreased the cytotoxic and cytopathic effects and intracellular replication of *P. salmonis* in the SHK-1 cell line against two *P. salmonis* isolates belonging to the EM90 genogroup (12201) and the LF89 genogroup (CGR02). There are few reports of experimental treatments against intracellular replication of *P. salmonis* in cell cultures, one of them being the one reported with quillay extracts, which contain mainly triterpene saponins [35]. However, the experimental design used in the experiments with quillay extracts is different from that used in this study with quercetin and silybin, since the authors used the CHSE-214 cell line to evaluate the intracellular replication of *P. salmonis*, a non-phagocytic cell line, unlike the SHK-1 cell line. In addition, they only evaluated the bacterial load of *P. salmonis* over 24 h of infection, while in our study, the bacterial load was evaluated after 7 and 14 days of infection, depending on the isolate. Quillay extracts have also been used to evaluate the effect of pre-incubation, co-incubation, and post-incubation on SHK-1 cells against infection with *P. salmonis* [36]. The proposed mechanism of action for these extracts is to promote phage–lysosome fusion. However, due to experimental differences with quercetin and silybin, the mechanisms of action by which these two compounds decrease the intracellular replication of *P. salmonis* could be different [36]. On the other hand, pre-incubation of quercetin and silybin in SHK-1 cells for 24 h prior to infection did not reduce the cytopathic effect of *P. salmonis* nor decrease the intracellular replication of the bacteria. These results differ from the effect reported for quillay extracts [36] and other intracellular pathogens such as *Salmonella typhimuirum* [37] and *Brucella abortus* [38], where pre-incubation with the treatments caused a reduction in the internalization and replication of the bacteria in the cell lines. However, the chemical structures of the compounds are different and the infection protocols are different, so the effect on the internalization and replication of intracellular bacteria is not necessarily through the same mechanisms of action, such as the activation of phage–lysosome fusion or a stimulation of the immune response [39,40,41]; however, further research is needed to elucidate the mechanism of action of quercetin and silybin. The last protocol developed in this study was the post-incubation of the compounds, which showed a decrease in the cytopathic effect and intracellular replication of *P. salmonis* but to a lesser extent than the co-incubation protocol. This result reinforces the idea that quercetin and silybin decrease the replication of *P. salmonis*, mediated by the interaction of the cell, bacteria, and compounds. On the other hand, the effects of quercetin and silybin were different, which could also demonstrate a difference between the mechanisms of the compounds in decreasing the internalization and/or intracellular replication of *P. salmonis*.

Finally, the results obtained from the three treatments show that the interaction between SHK-1 cells, *P. salmonis*, and the compounds is essential for the effect of quercetin and silybin in decreasing the intracellular replication of *P. salmonis*. This effect could be due to the ability of polyphenols to prevent or inhibit virulence factors through interactions with proteins found in the cytoplasmic membranes of bacteria that catalyze cell assembly and are important for the establishment of infection. In general, these interactions may be due to the ability of polyphenols to interact with bacterial proteins through different types of bonds such as covalent bonds [42,43], non-covalent bonds [44], and, mainly through hydrogen bonding interaction between polypeptide chains and polyphenols, which are generated between the hydroxyl groups of polyphenols and the polar groups carbonyl C = O and amino –NH_2_ of proteins [45,46], as well as hydrophobic interactions between polyphenols and proteins [47]. Both compounds have five hydroxyl groups, which could promote the formation of hydrogen bonds between the compounds and proteins of *P. salmonis* or SHK-1 cells, interfering with the bacteria/cell interaction. However, in silybin, only four of the hydroxyls are phenolic, which can alter the strength of the bond with proteins through these interactions. On the other hand, quercetin, unlike silybin, has a pyrane ring with a double bond between C2 and C3, which gives planarity and rigidity to the molecule. Studies with various enzymes in humans, such as amylase and lipase, have shown that the flat structure of the C ring, due to the presence of this double bond, increases the enzymatic inhibitory capacity of flavonoids. This structural feature seems decisive for the accommodation of flavonoids in their binding site on the protein, mainly through pi-stacking interactions [48,49,50,51]. Moreover, silybin has a structure with three benzene rings, so it would have a greater capacity to generate hydrophobic interactions (pi-stacking) with proteins compared to quercetin, as well as facilitating the passage between cell membranes. These differences between both molecules could be part of the reason why they present differences in their effects on the intracellular replication of *P. salmonis* in SHK-1 cells. Similar results have been reported for quercetin, where it has been shown to cause a decrease in the adhesion of *E. coli* O157:H7 in Caco-2 cells [52] and the adhesion of *L. monocytogenes* on steel surfaces [53,54]. Although there are several proposals related to anti-adhesion mechanisms, it is suggested that flavones, such as quercetin, would form a complex with the components of the cell wall, consequently inhibiting adhesion [7]. Besides, silybin has been observed to decrease the adhesion of *Staphylococcus epidermis* ATCC 35984 [55], although there are few studies on this subject. Interestingly, the protective effect of the compounds was not observed when the experiment was performed in RTgutGC cells. This was probably due to the fact that the mechanism of recognition of *P. salmonis* by these cells was either through other proteins or by a mechanism other than phagocytosis. Considering that co-incubation with quercetin and silybin could only decrease the replication of *P. salmonis* in phagocytic cells, the capacity of these compounds to decrease phagocytosis in SHK-1 cells was evaluated. The results related to the co-incubation with quercetin and silybin showed that both compounds decreased the phagocytosis of SHK-1 cells, but only when they were co-incubated in the cells, suggesting a possible mechanism by which these compounds may protect cells. However, the effects of both compounds were different; in the case of silybin, a dose-dependent effect was observed, both in the protection against infection with *P. salmonis* and in the decrease in the phagocytic capacity of SHK-1 cells. Similar results were reported in RAW 264.7 cells when treated with silybin and LPS, where a decrease in the phagocytic capacity of these cells was observed in a dose- and time-dependent manner. The possible mechanism described involves a possible inactivation of the MAPK, ER1/2, and RSK1/2 pathways [56]. In the case of quercetin, it has also been observed that in RAW 264.7 cells it decreases the phagocytosis of *Candida albicans* [57]. Other studies also report the effect of quercetin and silybin in decreasing phagocytic activity in different study models [58,59]. Future studies will be necessary to clarify the mechanism of action by which both compounds are able to reduce the replication of *P. salmonis* in phagocytic cells. Furthermore, considering the promising results in cell culture, it is necessary to evaluate whether these compounds can be used as treatments in salmonids against infections with *P. salmonis*.

## 4. Materials and Methods

### 4.1. Compounds, Cells and Bacteria

Silybin (98% purity) and quercetin (>95% purity) were commercially obtained from MERCK. The bacteria *P. salmonis* LF89-like isolate CGRO2 and *P. salmonis* EM90-like isolate 12201 were obtained from the Bioinformatics and Gene Expression Laboratory, Instituto de Nutricion y Tecnología de Alimentos (INTA); University of Chile. The cell lines used in this study were: the SHK-1 cell line obtained from the Virology Laboratory, Aquaculture Biotechnology Center, University of Santiago de Chile and the RTgutGC cell line obtained from the Comparative Immunology Laboratory, Aquaculture Biotechnology Center, University of Santiago de Chile.

### 4.2. Antibacterial Activity

To determine the MIC and IC_50_ of silybin and quercetin on two *P. salmonis* isolates, the microdilution method was performed with some modifications, as previously reported [60].

### 4.3. Cytotoxicity Assay

SHK-1 and RTgutGC cells were cultured in L-15 medium (Cytiva, Hyclone, South Logan, UT, USA) supplemented with 10% fetal bovine serum (Cytiva, Hyclone, South Logan, UT, USA), 4 mM L-glutamine (Mediatech, Corning, Manassas, VA, USA), and 40 μM β-mercaptoethanol (Life technologies, Gibco, New York, NY, USA) in T175 cell culture bottles (SPL). Then, 1 × 10^5^ cells were seeded in 24-well plates (SPL) for 24 h at 16 °C. Subsequently, the cell lines were incubated with three concentrations of quercetin and silybin (corresponding to the IC_50_, 1/2 IC_50_, and 1/4 IC_50_) for 24 h at 16 °C. After incubation, cells were detached from the plates using 50 μL of TripLE express (Life technologies, Gibco, New York, NY, USA) for 2 min; then, 450 μL of cell culture medium was added, and the cells were collected and centrifuged for 10 min at 1000× *g* at 4 °C. The resulting pellet was resuspended in 300 μL of Immunofluorescence (IF) buffer (Phosphate buffered saline PBS 1X, Corning and 2% Fetal Bovine Serum FBS, Cytiva) containing 1 μL of propidium iodide (1 mg/mL) (ChemCruz, Santa Cruz Biotechnology, Dallas, TX, USA),. Cell viability was determined using flow cytometry (BD FACSCanto II cytometer, BD Biosciences, Franklin Lakes, NJ, USA), considering IP-negative cells as viable [61].

### 4.4. Infection Assay in SHK-1 and RTgutGC Cell Line

Infection experiments in SHK-1 and RTgutGC cell lines were performed with isolates 12201 (like-EM90) and/or CGR02 (like-LF89), with an MOI (multiplicity of infection) of 50 and/or an MOI of 200 depending on the experiment. Then, 1 × 10^5^ cells were seeded in 24-well plates (SPL, Life sciences, Seoul, Republic of Korea) for 24 h at 16 °C. Subsequently, depending on the experiment, different infection protocols were performed. Infections performed in SHK-1 cells with *P. salmonis* 12201 were maintained for 7 days, while infections performed with *P. salmonis* CGR02 were maintained for 14 days. On the other hand, infections performed in the RTgutGC cell line with *P. salmonis* 12201 were maintained for 14 days. The cytopathic effect of *P. salmonis* infections in the different protocols mentioned above was observed at the end of the experiment using an AE2000 inverted microscope (Motic, China). The infection supernatant was collected, and the cells still adhered to the plate were collected by incubation for 5 min with TrypLE^TM^ Express (1X) (Life technologies, Gibco, New York, NY, USA); then, 450 μL of L-15 medium was added. The samples were stored at −20 °C for subsequent DNA extraction. The analysis of the cytotoxic effect of *P. salmonis* on cell cultures was performed using flow cytometry as previously explained; after the infection period, the cells adhered to the plate were collected. The experiments were performed in triplicate.

#### 4.4.1. Co-Infection Protocol

In the co-infection protocol (co), the cells were incubated at the same time with *P. salmonis* and the different treatments for 24 h at 16 °C. Subsequently, the cells were washed with 200 μL of 1X PBS (Mediatech, Corning, Manassas, VA, USA) and incubated for 1 h with L-15 medium containing 5% fetal bovine serum (Cytiva, Hyclone, South Logan, UT, USA) supplemented with 50 μg/mL of gentamicin. Finally, the cells were washed with 200 μL of 1X PBS (Mediatech, Corning, Manassas, VA, USA) and incubated again with L-15 medium containing 5% fetal bovine serum (Cytiva, Hyclone, South Logan, UT, USA) for 7 or 14 days depending on the isolate.

#### 4.4.2. Pre-Infection Protocol

In the pre-incubation protocol (pre), cells were incubated for 24 h at 16 °C with the different treatments, then washed with 200 μL of 1X PBS (Mediatech, Corning, Manassas, VA, USA), infected with *P. salmonis*, and incubated for 24 h at 16 °C. Subsequently, cells were washed with 200 μL of 1X PBS (Mediatech, Corning, Manassas, VA, USA) and incubated for 1 h with L-15 medium containing 5% fetal bovine serum (Cytiva, Hyclone, South Logan, UT, USA) supplemented with 50 μg/mL of gentamicin. They were then washed with 200 μL of 1X PBS (Mediatech, Corning, Manassas, VA, USA) and incubated again with L-15 medium containing 5% fetal bovine serum (Cytiva, Hyclone, South Logan, UT, USA) for 7 or 14 days depending on the isolate.

#### 4.4.3. Post-Infection Protocol

In the post-incubation protocol (post), cells were infected with *P. salmonis* and incubated for 24 h at 16 °C. Then, they were washed with 200 μL of 1X PBS (Mediatech, Corning, Manassas, VA, USA) and incubated for 1 h with L-15 medium containing 5% fetal bovine serum (Cytiva, Hyclone, South Logan, UT, USA) supplemented with 50 μg/mL of gentamicin; then, they were washed with 200 μL of 1X PBS (Mediatech, Corning, Manassas, VA, USA) and incubated for 24 h at 16 °C with the different treatments. Subsequently, the cells were washed with 200 μL of 1X PBS (Mediatech, Corning, Manassas, VA, USA) and incubated again with L-15 medium containing 5% fetal bovine serum (Cytiva, Hyclone, South Logan, UT, USA) for 7 or 14 days depending on the isolate.

### 4.5. DNA Extraction

DNA extraction was performed using 300 µL of the sample, 70 µL of 5X A solution (TRIS 279 mM, EDTA 101 mM, SDS 45 mM, β- mercaptoethanol 1.3% *v*/*v*, and NaCl 684 mM), and 4 µL of proteinase K (20 mg/mL, US Biological, Salem, MA, USA), which were incubated at 50 °C for 1 h. Subsequently, the samples were cooled to room temperature for 5 min, and 200 µL of basic saturated phenol (Winkler, Lampa, Chile) and 200 µL of chloroform (Winkler, Lampa, Chile) were added, shaken for 10 s using a vortex, and centrifuged for 5 min at 7000× *g*. The aqueous phase was collected and precipitated with 1 mL of cold absolute ethanol, incubating for 12 h at −20 °C. Samples were centrifuged for 30 min at 13,000× *g*, dried for 15 min, and resuspended in 30 µL of nuclease-free water (Apex, Genesee Scientific, El cajon, CA, USA).

### 4.6. Quantification by qPCR

The quantification of the bacterial load was carried out by detecting the *glyA* gene of *P. salmonis*. Reactions were performed in 96-well plates (Thermoscientific, Waltham, MA, USA) using the pikoReal 96 real-time PCR system (Thermoscientific). The reaction mixture was composed of 5 µL of SsoAdvanced Universal^TM^ SYBR^®^ Green Supermix (Biorad, South Granville, Australia), 0.5 µL of each primer (10 uM) (Fw 5′-GACTCGCGTACCATT GCAGA-3′ and Rv 5′-GCACACGCGGACTCGTATAA-3′) [62], 1 µL of DNA (50 ng), and 3 µL of ultrapure water (Apex, Genesee Scientific, El cajon, CA, USA), for a total of 10 µL. The thermal profile used was 1 cycle at 95 °C for 2 min, 35 cycles at 95 °C for 5 s, 61 °C for 15 s, and 72 °C for 15 s. To calculate the number of copies of the gene, a previously made calibration curve was employed for PCR amplification using Gotaq Green Master Mix (Progema, Madison, WI, USA). The reaction mix contains 12.5 µL of GoTaq^®^ Green Master Mix, 2X, 2.5 µL of each primer (10 µM), 1 µL of DNA, and 6.5 µL of nuclease-free water. The thermal profile used was 1 cycle at 95 °C for 2 min, 35 cycles at 95 °C for 15 s, 61 °C for 15 s, and 72 °C for 15 s, and a final cycle at 72 °C for 5 min. The PCR product obtained was purified using Wizard SV Gel and a PCR Clean-Up System Kit (Promega, Madison, WI, USA). Subsequently, dilutions from 10^2^ to 10^7^ were performed to obtain the calibration curve [63].

### 4.7. Phagocytocis Assay

The phagocytosis assay was performed on the SHK-1 cell line, for which 1 × 10^5^ cells were seeded in 24-well plates (SPL) for 24 h at 16 °C. Subsequently, the cell lines were incubated with two different protocols. In the first protocol, called co-incubation (co), the cells were incubated at the same time with 5 × 10^6^ carboxylate-modified FluoSpheres (Invitrogen, Eugene, OR, USA) and with the different treatments for 24 h at 16 °C. Then, the cells were washed 3 times with 200 μL of PBS 1X (Mediatech, Corning, Manassas, VA, USA) and were collected by incubation for 5 min with TrypLE^TM^ Express (1X) (Life technologies, Gibco, New York, NY, USA. Subsequently, 450 μL of L-15 medium was added, the cells were centrifuged at 1000× *g* for 5 min at 4 °C, and the cell pellet was resuspended in 300 μL of IF buffer PBS 1X, Corning and 2% FBS). On the other hand, in the second protocol, called pre-incubation (pre), the cells were incubated with the compounds for 24 h at 16 °C, and subsequently the cells were washed with 200 μL of 1X PBS (Mediatech, Corning, Manassas, VA, USA) and incubated with 5 × 10^6^ carboxylate-modified FluoSpheres (Invitrogen, Eugene, OR, USA) for 24 h at 16 °C. Then, the cells were washed 3 times with 200 μL of PBS 1X (Mediatech, Corning, Manassas, VA, USA) and collected by incubation for 5 min with TrypLE™ Express (1X) (Life technologies, Gibco, New York, NY, USA). Subsequently, 450 μL of L-15 medium was added and the cells were centrifuged at 1000× *g* for 5 min at 4 °C; then, the cell pellet was resuspended in 300 μL of IF buffer (PBS 1X, Corning and 2% FBS) containing 1 μL of propidium iodide (1 mg/mL) (ChemCruz, Santa Cruz Biotechnology, Dallas, TX, USA). Phagocytosis of the microspheres was detected using flow cytometry (BD FACSCanto II cytometer), considering the IP-negative and FITC-positive populations.

### 4.8. Statical Analysis

Statistical analyses were performed using GraphPad Prism 8.0. First, a test of normality and homogeneity of variance was performed and, depending on the result obtained, a two-way nonparametric Mann–Whitney test or a two-way parametric Welch’s test was applied to the data. Statistically significant differences were represented by stars, while the absence of statistically significant differences was represented by the letters ns. Multiple comparison analyses were performed using ANOVA tests with Welch’s correction. Statistically significant differences were represented by different letters.

## 5. Conclusions

Quercetin and silybin, two polyphenolic compounds, are able to decrease the intracellular replication of *P. salmonis* in SHK-1 cells when co-incubated at sub-inhibitory concentrations for 24 h; however, these compounds are not able to decrease the intracellular replication of *P. salmonis* in RTgutGC cells. Similarly, both compounds decreased the phagocytic capacity of SHK-1 cells in co-incubation. These results could indicate that the compounds affect the phagocytosis process of phagocytic cells, which are the target cells of *P. salmonis*. Future experiments are necessary to elucidate the mechanism by which these compounds have this effect, which could be an interaction with proteins of the bacteria, of the cells, or both. Finally, these results are promising to evaluate the effect of the administration of these compounds in *S. salar* against a challenge with *P. salmonis* as a possible treatment.

## 6. Patents

The research presented in this study is part of a patent application entitled “FOOD ADDITIVE TO COMBAT INFECTIOUS DISEASES CAUSED BY MARINE BACTERIAL PATHOGENS” Application number 202402559.

## Figures and Tables

**Table 1:** MIC and IC_50_ values calculated for quercetin and silybin on four *P. salmonis* isolates.

	*P. salmonis* CGR02		*P. salmonis* 12201	
Compound	MIC µg/mL	IC_50_ µg/mL	MIC µg/mL	IC_50_ µg/mL
Quercetin	38.6 ± 7.6	26.8 ± 2.6	62.7 ± 16.5	30.2 ± 7.7
Silybin	134.3 ± 1.9	71.0 ± 17.4	132.5 ± 0.8	74.0 ± 6.5

**Table 2:** Sub-inhibitory concentration values of quercetin and silybin used for cell culture experiments.

Compound	IC_50_ µg/mL	½ IC_50_ µg/mL	¼ IC_50_ µg/mL
Quercetin	27 (Q)	14 (Q/2)	7 (Q/4)
Silybin	68 (S)	34 (S/2)	17 (S/4)

## Data Availability

The raw data supporting the conclusions of this article will be made available by the authors on request.

## References

[1] FAO (2022). The State of World Fisheries and Aquaculture 2022.

[2] Sernapesca (2023). Informe Sanitario Con Información Sanitaria de Agua Dulce y Mar.

[3] Maisey K., Montero R., Christodoulides M. (2017). Vaccines for Piscirickettsiosis (Salmonid Rickettsial Septicaemia, SRS): The Chile Perspective. Expert. Rev. Vaccines.

[4] Sernapesca (2023). Informe Sobre Uso de Antimicrobianos en la Salmonicultura Nacional.

[5] Hossain A., Habibullah-Al-Mamun M., Nagano I., Masunaga S., Kitazawa D., Matsuda H. (2022). Antibiotics, Antibiotic-Resistant Bacteria, and Resistance Genes in Aquaculture: Risks, Current Concern, and Future Thinking. Environ. Sci. Pollut. Res..

[6] Quiñones R.A., Fuentes M., Montes R.M., Soto D., León-Muñoz J. (2019). Environmental Issues in Chilean Salmon Farming: A Review. Rev. Aquac..

[7] Farhadi F., Khameneh B., Iranshahi M., Iranshahy M. (2019). Antibacterial Activity of Flavonoids and Their Structure–Activity Relationship: An Update Review. Phytother. Res..

[8] Duda-Madej A., Stecko J., Sobieraj J., Szymańska N., Kozłowska J. (2022). Naringenin and Its Derivatives—Health-Promoting Phytobiotic against Resistant Bacteria and Fungi in Humans. Antibiotics.

[9] Elbatreek M.H., Mahdi I., Ouchari W., Mahmoud M.F., Sobeh M. (2023). Current Advances on the Therapeutic Potential of Pinocembrin: An Updated Review. Biomed. Pharmacother..

[10] Klančnik A., Šimunović K., Kovac J., Sahin O., Wu Z., Vučković D., Abram M., Zhang Q., Možina S.S. (2019). The Anti-Campylobacter Activity and Mechanisms of Pinocembrin Action. Microorganisms.

[11] Echeverría J., Opazo J., Mendoza L., Urzúa A., Wilkens M. (2017). Structure-Activity and Lipophilicity Relationships of Selected Antibacterial Natural Flavones and Flavanones of Chilean Flora. Molecules.

[12] Šmejkal K., Chudík S., Klouček P., Marek R., Cvačka J., Urbanová M., Julínek O., Kokoška L., Šlapetová T., Holubová P. (2008). Antibacterial C-Geranylflavonoids from *Paulownia tomentosa* Fruits. J. Nat. Prod..

[13] Dzoyem J.P., Melong R., Tsamo A.T., Tchinda A.T., Kapche D.G.W.F., Ngadjui B.T., McGaw L.J., Eloff J.N. (2017). Cytotoxicity, Antimicrobial and Antioxidant Activity of Eight Compounds Isolated from *Entada abyssinica* (Fabaceae). BMC Res. Notes.

[14] Escandón R.A., del Campo M., López-Solis R., Obreque-Slier E., Toledo H. (2016). Antibacterial Effect of Kaempferol and (−)-Epicatechin on *Helicobacter pylori*. Eur. Food Res. Technol..

[15] Eerdunbayaer, Orabi M.A.A., Aoyama H., Kuroda T., Hatano T. (2014). Structures of Two New Flavonoids and Effects of *Licorice phenolics* on Vancomycin-Resistant *Enterococcus* Species. Molecules.

[16] Defoirdt T. (2013). Antivirulence Therapy for Animal Production: Filling an Arsenal with Novel Weapons for Sustainable Disease Control. PLoS Pathog..

[17] Ben Lagha A., Haas B., Grenier D. (2017). Tea Polyphenols Inhibit the Growth and Virulence Properties of *Fusobacterium nucleatum*. Sci. Rep..

[18] Li R., Lu J., Duan H., Yang J., Tang C. (2020). Biofilm Inhibition and Mode of Action of *Epigallocatechin gallate* against *Vibrio mimicus*. Food Control.

[19] Rasko D.A., Sperandio V. (2010). Anti-Virulence Strategies to Combat Bacteria-Mediated Disease. Nat. Rev. Drug Discov..

[20] Bhattacharya D., Sinha R., Mukherjee P., Howlader D.R., Nag D., Sarkar S., Koley H., Withey J.H., Gachhui R. (2020). Anti-Virulence Activity of Polyphenolic Fraction Isolated from Kombucha against *Vibrio cholerae*. Microb. Pathog..

[21] Çevikbaş H., Ulusoy S., Kaya Kinaytürk N. (2024). Exploring Rose Absolute and Phenylethyl Alcohol as Novel Quorum Sensing Inhibitors in *Pseudomonas aeruginosa* and *Chromobacterium violaceum*. Sci. Rep..

[22] Barbosa P.d.P.M., Ruviaro A.R., Martins I.M., Macedo J.A., LaPointe G., Macedo G.A. (2021). Enzyme-Assisted Extraction of Flavanones from Citrus Pomace: Obtention of Natural Compounds with Anti-Virulence and Anti-Adhesive Effect against *Salmonella enterica* subsp. Enterica Serovar Typhimurium. Food Control.

[23] Esteban-Fernández A., Zorraquín-PenÌa I., Ferrer M.D., Mira A., Bartolomé B., González De Llano D., Victoria Moreno-Arribas M. (2018). Inhibition of Oral Pathogens Adhesion to Human Gingival Fibroblasts by Wine Polyphenols Alone and in Combination with an Oral Probiotic. J. Agric. Food Chem..

[24] Rojas V., Galanti N., Bols N.C., Marshall S.H. (2009). Productive Infection of *Piscirickettsia salmonis* in Macrophages and Monocyte-like Cells from Rainbow Trout, a Possible Survival Strategy. J. Cell Biochem..

[25] Gómez F.A., Tobar J.A., Henríquez V., Sola M., Altamirano C., Marshall S.H. (2013). Evidence of the Presence of a Functional Dot/Icm Type IV-B Secretion System in the Fish Bacterial Pathogen *Piscirickettsia salmonis*. PLoS ONE.

[26] Levipan H.A., Irgang R., Opazo L.F., Araya-León H., Avendaño-Herrera R. (2022). Collective Behavior and Virulence Arsenal of the Fish Pathogen *Piscirickettsia salmonis* in the Biofilm Realm. Front. Cell Infect. Microbiol..

[27] Marshall S.H., Gómez F.A., Ramírez R., Nilo L., Henríquez V. (2012). Biofilm Generation by *Piscirickettsia salmonis* under Growth Stress Conditions: A Putative in Vivo Survival/Persistence Strategy in Marine Environments. Res. Microbiol..

[28] Santibáñez N., Vega M., Pérez T., Enriquez R., Escalona C.E., Oliver C., Romero A. (2024). In Vitro Effects of Phytogenic Feed Additive on *Piscirickettsia salmonis* Growth and Biofilm Formation. J. Fish. Dis..

[29] Figueroa J., Villagrán D., Cartes C., Solis C., Nourdin-Galindo G., Haussmann D. (2021). Analysis of Genes Encoding for Proteolytic Enzymes and Cytotoxic Proteins as Virulence Factors of *Piscirickettsia salmonis* in SHK-1 Cells. J. Fish. Dis..

[30] Śanchez P., Oliver C., Herńandez M., Cortés M., Cecilia Rauch M., Valenzuela K., Garduno R.A., Avendano-Herrera R., Yánez A.J. (2018). In Vitro Genomic and Proteomic Evidence of a Type IV Pili-like Structure in the Fish Pathogen *Piscirickettsia salmonis*. FEMS Microbiol. Lett..

[31] Carril G.P., Gómez F.A., Marshall S.H. (2017). Expression of Flagellin and Key Regulatory Flagellar Genes in the Non-Motile Bacterium *Piscirickettsia salmonis*. Dis. Aquat. Organ..

[32] Ortiz-Severín J., Travisany D., Maass A., Cambiazo V., Chávez F.P. (2020). Global Proteomic Profiling of *Piscirickettsia salmonis* and Salmon Macrophage-like Cells during Intracellular Infection. Microorganisms.

[33] Shamsudin N.F., Ahmed Q.U., Mahmood S., Shah S.A.A., Khatib A., Mukhtar S., Alsharif M.A., Parveen H., Zakaria Z.A. (2022). Antibacterial Effects of Flavonoids and Their Structure-Activity Relationship Study: A Comparative Interpretation. Molecules.

[34] Rozas-Serri M. (2022). Why Does *Piscirickettsia salmonis* Break the Immunological Paradigm in Farmed Salmon? Biological Context to Understand the Relative Control of Piscirickettsiosis. Front. Immunol..

[35] Cañon-Jones H., Cortes H., Castillo-Ruiz M., Schlotterbeck T., Martín R.S. (2020). *Quillaja saponaria* (Molina) Extracts Inhibits in Vitro *Piscirickettsia salmonis* Infections. Animals.

[36] Cortés H.D., Gómez F.A., Marshall S.H. (2021). The Phagosome–Lysosome Fusion Is the Target of a Purified *Quillaja saponaria* Extract (Pqse) in Reducing Infection of Fish Macrophages by the Bacterial Pathogen *Piscirickettsia salmonis*. Antibiotics.

[37] Birhanu B.T., Lee E.B., Lee S.J., Park S.C. (2021). Targeting *Salmonella typhimurium* Invasion and Intracellular Survival Using Pyrogallol. Front. Microbiol..

[38] Arayan L.T., Simborio H.L., Reyes A.W.B., Hop H.T., Min W.G., Lee H.J., Rhee M.H., Chang H.H., Kim S. (2015). The Effects of Red Ginseng Saponin Fraction-A (RGSF-A) on Phagocytosis and Intracellular Signaling in Brucella Abortus Infected RAW 264.7 Cells. FEMS Microbiol. Lett..

[39] Adrar N.S., Madani K., Adrar S. (2019). Impact of the Inhibition of Proteins Activities and the Chemical Aspect of Polyphenols-Proteins Interactions. PharmaNutrition.

[40] Da Cunha L.R., Muniz-Junqueira M.I., Dos Santos Borges T.K. (2019). Impact of Polyphenols in Phagocyte Functions. J. Inflamm. Res..

[41] Wang S., Li Z., Ma Y., Liu Y., Lin C.C., Li S., Zhan J., Ho C.T. (2021). Immunomodulatory Effects of Green Tea Polyphenols. Molecules.

[42] Jongberg S., Andersen M.L., Lund M.N. (2020). Covalent Protein-Polyphenol Bonding as Initial Steps of Haze Formation in Beer. J. Am. Soc. Brew. Chem..

[43] Yang C., Wang B., Wang J., Xia S., Wu Y. (2019). Effect of Pyrogallic Acid (1,2,3-Benzenetriol) Polyphenol-Protein Covalent Conjugation Reaction Degree on Structure and Antioxidant Properties of Pumpkin (*Cucurbita* sp.) Seed Protein Isolate. LWT.

[44] Achika J.I., Ayo R.G., Oyewale A.O., Habila J.D. (2020). Flavonoids with Antibacterial and Antioxidant Potentials from the Stem Bark of *Uapaca heudelotti*. Heliyon.

[45] Quan T.H., Benjakul S., Sae-leaw T., Balange A.K., Maqsood S. (2019). Protein–Polyphenol Conjugates: Antioxidant Property, Functionalities and Their Applications. Trends Food Sci. Technol..

[46] Save S.N., Choudhary S. (2018). Elucidation of Energetics and Mode of Recognition of Green Tea Polyphenols by Human Serum Albumin. J. Mol. Liq..

[47] Zhao L., Zhou A., Liu Z., Xiao J., Wang Y., Cao Y., Wang L. (2020). Inhibitory Mechanism of Lactoferrin on Antibacterial Activity of Oenothein B: Isothermal Titration Calorimetry and Computational Docking Simulation. J. Sci. Food Agric..

[48] Martinez-Gonzalez A.I., Díaz-Sánchez G., de la Rosa L.A., Bustos-Jaimes I., Alvarez-Parrilla E. (2019). Inhibition of α-Amylase by Flavonoids: Structure Activity Relationship (SAR). Spectrochim. Acta A Mol. Biomol. Spectrosc..

[49] Martinez-Gonzalez A.I., Díaz-Sánchez Á.G., De La Rosa L.A., Vargas-Requena C.L., Bustos-Jaimes I., Alvarez-Parrilla E. (2017). Polyphenolic Compounds and Digestive Enzymes: In Vitro Non-Covalent Interactions. Molecules.

[50] Martinez-Gonzalez A.I., Alvarez-Parrilla E., Díaz-Sánchez Á.G., de la Rosa L.A., Núñez-Gastélum J.A., Vazquez-Flores A.A., Gonzalez-Aguilar G.A. (2017). In Vitro Inhibition of Pancreatic Lipase by Polyphenols: A Kinetic, Fluorescence Spectroscopy and Molecular Docking Study. Food Technol. Biotechnol..

[51] Lo Piparo E., Scheib H., Frei N., Williamson G., Grigorov M., Chou C.J. (2008). Flavonoids for Controlling Starch Digestion: Structural Requirements for Inhibiting Human α-Amylase. J. Med. Chem..

[52] Xue Y., Du M., Zhu M.J. (2019). Quercetin Prevents *Escherichia coli* O157:H7 Adhesion to Epithelial Cells via Suppressing Focal Adhesions. Front. Microbiol..

[53] Vazquez-Armenta F.J., Hernandez-Oñate M.A., Martinez-Tellez M.A., Lopez-Zavala A.A., Gonzalez-Aguilar G.A., Gutierrez-Pacheco M.M., Ayala-Zavala J.F. (2020). Quercetin Repressed the Stress Response Factor (SigB) and Virulence Genes (PrfA, ActA, InlA, and InlC), Lower the Adhesion, and Biofilm Development of *L. Monocytogenes*. Food Microbiol..

[54] Vazquez-Armenta F.J., Bernal-Mercado A.T., Tapia-Rodriguez M.R., Gonzalez-Aguilar G.A., Lopez-Zavala A.A., Martinez-Tellez M.A., Hernandez-Oñate M.A., Ayala-Zavala J.F. (2018). Quercetin Reduces Adhesion and Inhibits Biofilm Development by *Listeria monocytogenes* by Reducing the Amount of Extracellular Proteins. Food Control.

[55] Evren E., Yurtcu E. (2015). In Vitro Effects on Biofilm Viability and Antibacterial and Antiadherent Activities of Silymarin. Folia Microbiol..

[56] Sun K.H., Lee M.Y., Jeon Y.J. (2023). Inhibition of Phagocytosis by Silibinin in Mouse Macrophages. Curr. Issues Mol. Biol..

[57] Cui S., Qian J., Bo P. (2013). Inhibitive Effect on Phagocytosis of *Candida albicans* Induced by Pretreatment with Quercetin via Actin Cytoskeleton Interference. J. Tradit. Chin. Med..

[58] Nickel T., Hanssen H., Sisic Z., Pfeiler S., Summo C., Schmauss D., Hoster E., Weis M. (2011). Immunoregulatory Effects of the Flavonol Quercetin In Vitro and In Vivo. Eur. J. Nutr..

[59] Tsai C.F., Chen G.W., Chen Y.C., Shen C.K., Lu D.Y., Yang L.Y., Chen J.H., Yeh W.L. (2022). Regulatory Effects of Quercetin on M1/M2 Macrophage Polarization and Oxidative/Antioxidative Balance. Nutrients.

[60] Parra M., Aldabaldetrecu M., Arce P., Soto-Aguilera S., Vargas R., Guerrero J., Tello M., Modak B. (2024). [Cu(NN1)_2_]ClO_4_, a Copper (I) Complex as an Antimicrobial Agent for the Treatment of Piscirickettsiosis in Atlantic Salmon. Int. J. Mol. Sci..

[61] Aldabaldetrecu M., Parra M., Soto S., Arce P., Tello M., Guerrero J., Modak B. (2020). New Copper(I) Complex with a Coumarin as Ligand with Antibacterial Activity against *Flavobacterium psychrophilum*. Molecules.

[62] Ortiz-Severín J., Travisany D., Maass A., Chávez F.P., Cambiazo V. (2019). *Piscirickettsia salmonis* Cryptic Plasmids: Source of Mobile DNA and Virulence Factors. Pathogens.

[63] Zúñiga A., Aravena P., Pulgar R., Travisany D., Ortiz-Severín J., Chávez F.P., Maass A., González M., Cambiazo V. (2020). Transcriptomic Changes of *Piscirickettsia salmonis* During Intracellular Growth in a Salmon Macrophage-Like Cell Line. Front. Cell Infect. Microbiol..

